# BIOPSYCHOSOCIAL RISK AND RESILIENCE PATHWAYS IN DEMENTIA INEQUALITIES

**DOI:** 10.1093/geroni/igac059.451

**Published:** 2022-12-20

**Authors:** Laura Zahodne

**Affiliations:** University of Michigan, Ann Arbor, Michigan, United States

## Abstract

In the United States, racial/ethnic inequalities in Alzheimer's disease and related dementias persist even after controlling for socioeconomic factors and physical health. Persistent and unexplained disparities suggest: (1) there are unrecognized dementia risk factors that are socially patterned and/or (2) known dementia risk factors exhibit differential impact across social groups. This talk will present data from multiple longitudinal studies of brain and cognitive aging to support each possibility. On average, marginalized racial/ethnic groups are more likely than non-Latinx Whites to experience structural and interpersonal discrimination, social and economic constraints, as well as barriers to accessing high quality education. However, these same groups also show evidence of greater psychosocial resilience that is linked to better late-life cognitive health. This talk will demonstrate how specific psychosocial factors can contribute to or offset dementia disparities, illustrate major challenges to this work, and introduce new data collection efforts to advance the field.

